# A Case of Unusual Presentation of Kartagener’s Syndrome in a 22-Year-Old Female Patient

**DOI:** 10.7759/cureus.28119

**Published:** 2022-08-17

**Authors:** Sweta Sahu, Ravishankar Ranganatha, Umesh Batura, Udit Choubey, Dasari Ragasri Meghana, Vyshnav R Menon, Mihirkumar P Parmar, Anup Banur, Deepanshu Raj, Harshith Manjunath

**Affiliations:** 1 Internal Medicine, Jagadguru Jayadeva Murugarajendra (JJM) Medical College, Davanagere, IND; 2 Internal Medicine, Karnataka Institute of Medical Sciences, Hubballi, IND; 3 Internal Medicine, American University of Antigua, St. John's, ATG; 4 Internal Medicine, Shyam Shah Medical College, Rewa, IND; 5 Internal Medicine, Kakatiya Medical College, Warangal, IND; 6 Internal Medicine, Washington University of Health and Science, San Pedro, BLZ; 7 Internal Medicine, Gujarat Medical Education and Research Society (GMERS) Medical College, Vadnagar, IND; 8 Pulmonology, SS Institute of Medical Sciences and Research Centre (SSIMS & RC), Davanagere, IND; 9 Internal Medicine, SS Institute of Medical Sciences and Research Centre (SSIMS & RC), Davanagere, IND; 10 Medicine, Jagadguru Jayadeva Murugarajendra (JJM) Medical College, Davanagere, IND

**Keywords:** young females, respiratory tract infections, situs inversus with dextrocardia, sinusitis, bronchiectasis

## Abstract

Kartagener’s syndrome (KS) is a rare hereditary disease. It is a triad of chronic sinusitis bronchiectasis and situs inversus. The condition is probably underdiagnosed and the symptoms are more prevalent in children in their first decade of life. We describe a case of a 22-year-old female with a history of cough and dyspnea for six months. The patient was diagnosed with dextrocardia at birth and had no significant medical history for the first two decades of her life. She was adequately immunized during her infancy and childhood as per the National Immunization Program. She was also vaccinated with the SARS-CoV-2 vaccine along with the booster dose. She was diagnosed with KS depending on her clinical symptoms, imaging characteristics and demographics. The patient had received symptomatic treatment for six months before developing respiratory distress requiring hospitalization when the diagnosis was made. KS has to be taken into consideration if an individual with dextrocardia has recurrent upper or lower respiratory tract infections. An early and accurate diagnosis of this illness is crucial to avoid complications and improve the quality of life of patients.

## Introduction

A wider class of ciliary motility illnesses known as primary ciliary dyskinesias (PCDs) includes Kartagener’s syndrome (KS) as a subgroup [1]. KS is a hereditary autosomal recessive illness described by deficiencies in the activity of ciliary movement and consists of triad bronchiectasis, situs inversus, and sinusitis [2]. In 1904, Siewert provided the first explanation of the association between bronchiectasis, chronic sinusitis, and situs inversus [3]. In 1933, Kartagener identified the congenital condition that bears his name as the first to detect this clinical triad [4]. Its occurrence is roughly 1 in 30,000 live births, according to estimates [5]. Normal ciliary activity is essential for sperm motility, host defense in the respiratory system, and appropriate visceral orientation throughout development. The DNAI1 and DNAH5 gene mutation in KS causes decreased ciliary motility, which increases the risk of infertility, left-right body alignment problems, and recurrent sinopulmonary infections [5]. Females and males are equally affected [6].

The instance of a 22-year-old female with KS is described in this case report owing to the late diagnosis and rarity of clinical presentation. The differential diagnosis of cystic fibrosis (CF) included consideration of this syndrome. In CF, pulmonary disease predominantly impacts the upper lobes, whereas in KS, and other primary ciliary dyskinesia, the middle and lower lobes are most affected. Sinus illness is prevalent in both primary ciliary dyskinesia and CF, however, nasal polyps are characteristic of CF. The objective of this study was to understand KS better and learn about the complications of KS when diagnosed late.

## Case presentation

A 22-year-old female patient was brought to the emergency department (ED) on July 6, 2022, with a history of dyspnea, cough with expectoration for six months which had worsened over the last three days, and fever for three days. There was no prior history of allergies or weight loss. There was no history of atopy or asthma in the family. Both parents were healthy and it was a non-consanguineous marriage and the pregnancy was uneventful. At birth, her mother reported that the pediatrician had mentioned that this patient had dextrocardia. 

Her siblings had no significant medical history. She had a history of productive cough and breathlessness for the past six months because of which she had visited the outpatient department of a primary care hospital five times and had received symptomatic treatment.

On presentation to ED, the patient was in respiratory distress, conscious, and oriented. On examination of her vitals, it was found that the patient was febrile with high respiratory and pulse rate and low oxygen saturation (Table 1).

**Table 1:** Vitals findings of the patient at the time of presentation SpO_2_: saturation of peripheral oxygen

Vitals	Value	Normal range	
Pulse rate (beats per minute)	134	60-100	
Respiratory rate (cycles per minute)	50	12-18	
SpO_2_ (%)	48	95 and above	
Blood pressure (mm Hg)	102/58	90/60 to 120/80	
Temperature (°F)	101.8	97.5-98.9	

She was started on supplemental oxygen with simple face mask at 10 L/min and was immediately transferred to ICU. Respiratory system examination revealed normal vesicular breath sounds bilaterally with bilateral rhonchi and coarse crackles on basal lung fields. Her apex beat was felt in the right fifth intercostal area along the midclavicular line. She had grade 3 clubbing in all digits. Rest of the examination was unremarkable. Her blood gas revealed hypercapnic respiratory failure with normal lactates (Table 2).

**Table 2:** Arterial blood gas analysis of patient at the time of admission PaO_2_: partial pressure of oxygen in arterial blood; PaCO_2_: partial pressure of carbon dioxide in arterial blood; HCO_3_^−^: bicarbonate ion

Parameters	Value	Normal range
pH	7.3	7.35-7.45
PaCO_2_ (mm Hg)	58	32-48
PaO_2_ (mm Hg)	56	83-108
HCO_3_^-^ (mmol/L)	28	22-26

**Figure 1:**
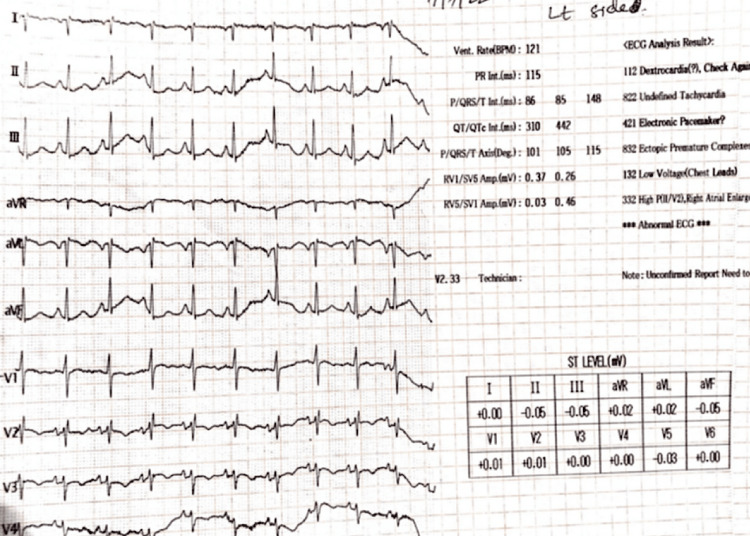
ECG findings from left-side chest leads aVR: augmented vector right; aVL: augmented vector left; aVF: augmented vector foot

**Figure 2:**
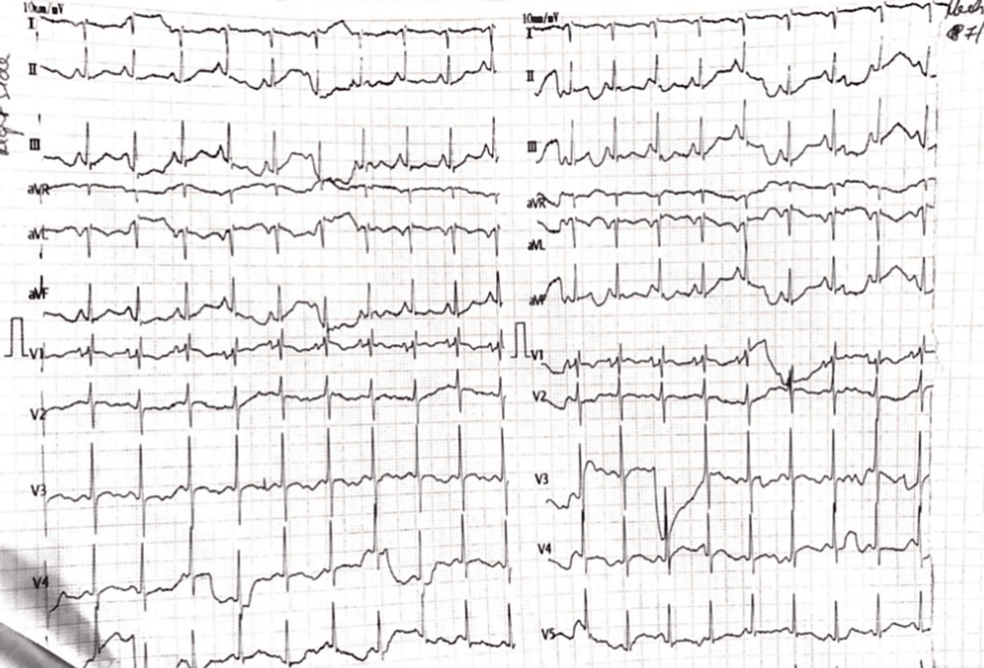
ECG findings from right-side chest leads aVR: augmented vector right; aVL: augmented vector left; aVF: augmented vector foot

The patient was put on non-invasive ventilation (NIV) and started on intravenous antibiotics, systemic corticosteroids, inhaled bronchodilators and mucolytics, chest physiotherapy, and other supportive treatment. A sputum analysis which included smear microscopy and Gene-Xpert was conducted to rule out other probable conditions. An electrocardiogram (ECG) was taken, which indicated inverted T waves in V3 and V4, negative P waves in lead 1 and aVF, and the axis is northwest (Figure 1). The ECG findings were normal for the right-sided chest leads (Figure 2). Blood investigations showed leukocytosis with neutrophilia. 

**Figure 3:**
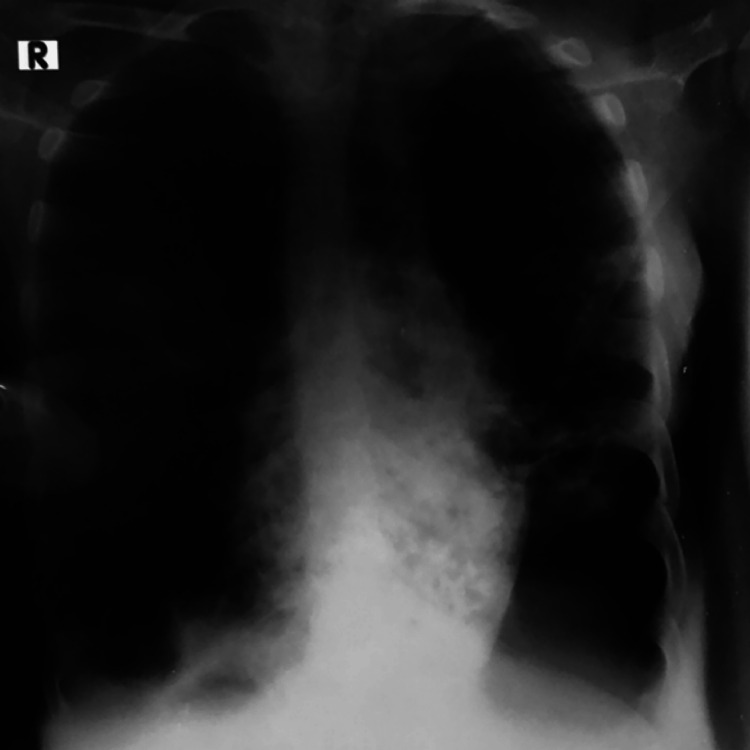
Chest x-ray of the patient showing bilateral bronchiectasis with cyst in the left lower zone and hyperinflated lower lung field

**Figure 4:**
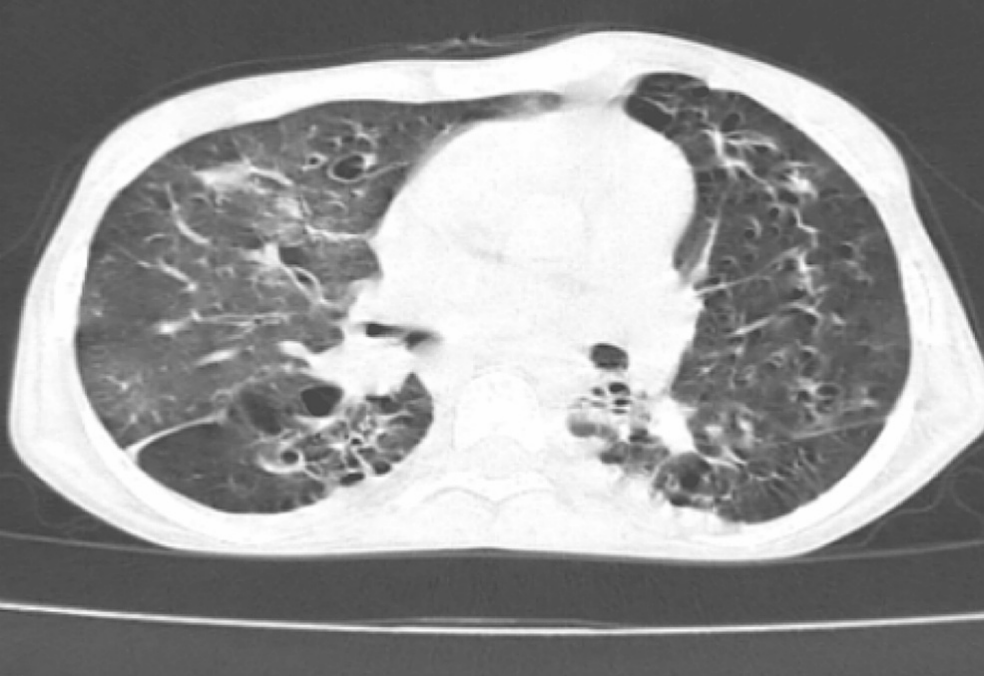
CT of the thorax showing co-existing emphysematous hyperinflation

**Figure 5:**
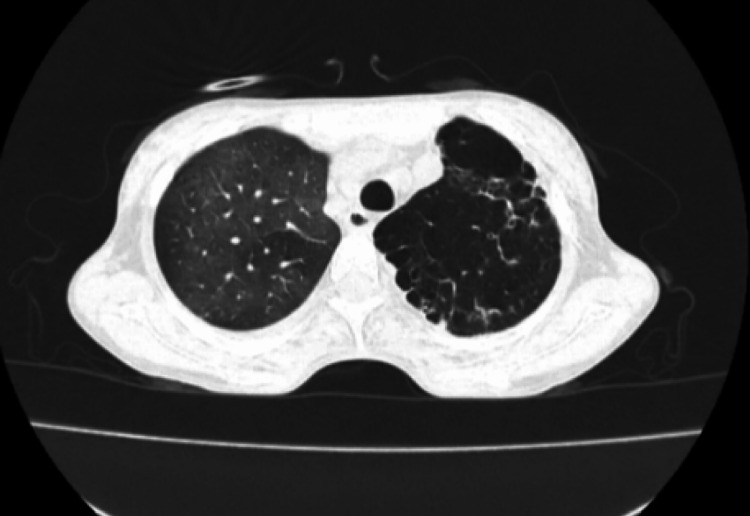
Computed tomography of the thorax

**Figure 6:**
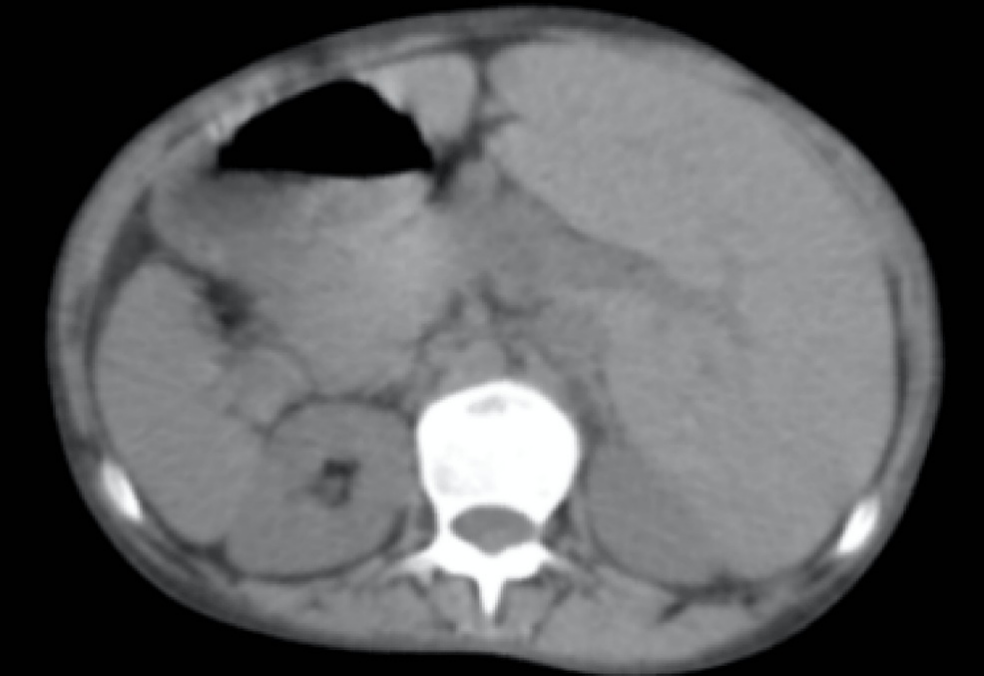
CT of the abdomen showing situs inversus

A bedside chest x-ray showed bilateral bronchiectasis with a cyst in the left lower zone and hyperinflated lower lung field (Figure 3). Once the patient was stabilized, further evaluation was done. CT thorax revealed situs inversus with the right-sided aorta and multifocal bronchiectasis involving both the lungs, predominantly in the lingual, right middle, and bilateral lower lobes. Minimal bronchiectasis was observed in the left upper lobe with emphysematous changes. Co-existing emphysematous hyperinflation was noted in the remaining part of the lower lobe (Figures 4, 5). The liver, as well as “inferior vena” cava, were on the left side of the CT scan of her abdomen and pelvis, while the spleen was on the right side, suggesting situs inversus (Figure 6). CT of her paranasal sinus revealed that she had minimal mucosal thickenings in ethmoid and sphenoid sinuses with a noted mucosal thickening in the maxillary sinus. The left frontal sinus showed well aeration and the maxillary sinus showed partial sinus. Then, KS was diagnosed based on clinical signs along with imaging characteristics. Two-dimensional echocardiography (2D ECHO) revealed that she had a dilated right atrium and right ventricle and severe pulmonary artery hypertension.

The patient gradually improved over three days and was weaned off non-invasive ventilation (NIV) and was shifted to ward on day four of admission. She was discharged on day seven after a pulmonology consult. She was advised for domiciliary oxygen therapy, oral antibiotics, and chest physiotherapy. She was also advised about the need for annual influenza, pneumococcal vaccination, and other necessary preventive measures.

## Discussion

Problems with ciliary motility could be acquired or inherited. Congenital conditions are known as primary ciliary dyskinesias (PCDs). Situs inversus is seen in about 50% of PCD patients. Such PCD instances with “situs inversus” are referred to as Kartagener's syndrome [1]. PCD’s clinical signs can vary; some may start with neonatal respiratory distress and later progress to chronic productive cough caused by bronchiectasis, atypical asthma that does not respond to treatment, chronic rhinosinusitis, otitis, subfertility, and ectopic pregnancy in females, or male infertility [7]. Radiographic and clinical signs of bronchiectasis appear as the condition develops; obstructive and bronchiectasis impairment might be seen in pre-schoolers [8]. In our case, the patient had been asymptomatic for two decades of her life and she survived the initial two years of the COVID-19 pandemic as well. Usually, the cases of KS and CF are diagnosed in neonates or in early childhood because of their recurrent pulmonary infections. Early diagnosis and regular clinical follow-ups are very crucial for these patients to avoid consequences since there is no definitive cure for such conditions. A late diagnosis of developed bronchiectasis worsens the prognosis, even with the most effective treatments. The complications could affect the quality of life of the patient to a great extent.

## Conclusions

Kartagener's syndrome and cystic fibrosis both are well diagnosed in early childhood with diagnostic symptoms. However, in some cases, KS can stay asymptomatic for a long time. The early detection of KS is crucial for the condition's overall prognosis, as many unknown complications can come forth due to late diagnosis. A late diagnosis can deteriorate the quality of a patient’s life and make symptomatic management more difficult than expected.
